# Collaborative interactions of heterogenous ribonucleoproteins contribute to transcriptional regulation of sterol metabolism in mice

**DOI:** 10.1038/s41467-020-14711-4

**Published:** 2020-02-20

**Authors:** Zhengyi Zhang, An-Chieh Feng, David Salisbury, Xin Liu, Xiaohui Wu, Jason Kim, Irina Lapina, Dan Wang, Brennan Lee, Josue Fraga, Calvin Pan, Kevin J. Williams, Aldons J. Lusis, Phil Scumpia, Tamer Sallam

**Affiliations:** 1 Division of Cardiology, Department of Medicine, University of California, Los Angeles, CA 90095 USA; 2 Molecular Biology Institute, University of California, Los Angeles, CA 90095 USA; 3 Molecular Biology Interdepartmental Doctoral Program, University of California, Los Angeles, CA 90095 USA; 4 Division of Dermatology, Department of Medicine, University of California, Los Angeles, CA 90095 USA; 5 Department of Microbiology, Immunology and Molecular Genetics, University of California, Los Angeles, 90095 USA; 6 Department of Human Genetics, University of California, Los Angeles, CA 90095 USA; 7 Department of Biological Chemistry, University of California, Los Angeles, CA 90095 USA; 8 Molecular, Cellular and Integrative Physiology Program, University of California, Los Angeles, CA 90095 USA

**Keywords:** Gene expression, Long non-coding RNAs, Transcription, Fat metabolism, Dyslipidaemias

## Abstract

Heterogeneous nuclear ribonucleoproteins (hnRNPs) are a group of functionally versatile proteins that play critical roles in the biogenesis, cellular localization and transport of RNA. Here, we outline a role for hnRNPs in gene regulatory circuits controlling sterol homeostasis. Specifically, we find that tissue-selective loss of the conserved hnRNP RALY enriches for metabolic pathways. Liver-specific deletion of RALY alters hepatic lipid content and serum cholesterol level. In vivo interrogation of chromatin architecture and genome-wide RALY-binding pattern reveal insights into its cooperative interactions and mode of action in regulating cholesterogenesis. Interestingly, we find that RALY binds the promoter region of the master metabolic regulator *Srebp2* and show that it directly interacts with coactivator Nuclear Transcription Factor Y (NFY) to influence cholesterogenic gene expression. Our work offers insights into mechanisms orchestrating selective promoter activation in metabolic control and a model by which hnRNPs can impact health and disease states.

## Abstract (web summary)

Heterogeneous nuclear ribonucleoproteins (hnRNPs) play critical roles in the biogenesis, localization and transport of RNA. Here authors investigate a role for hnRNPs in sterol metabolism in mice and provide insights into their role in selective promoter activation.

## Introduction

Heterogeneous nuclear ribonucleoproteins (hnRNPs) are a family of multifunctional RNA-binding proteins with critical roles in gene regulation. Cooperative activities of hnRNPs have been shown to impact various aspects of RNA metabolism^1^. Although much of the focus on hnRNPs has been geared to their role in splicing and transcript processing, their direct effects on mRNA biogenesis are far less understood with limited compelling roles for transcriptional contributions of hnRNPs. In addition, several lines of evidence link hnRNP abnormalities to neurodegenerative diseases and cancer, but their impact in metabolic control remains unexplored^2,3^.

A number of genome-wide association studies have linked variants at the hnRNP *RALY* (also known as heterogeneous nuclear ribonucleoprotein C-like 2) with cardiometabolic traits, including total cholesterol and coronary artery disease, yet little is known about the function and mechanisms of actions of RALY^4–6^. Our previous studies have shown that RALY interacts with *LeXis*, a noncoding RNA mediating crosstalk between the cholesterol biosynthesis and efflux pathways^7^. *LeXis* is a direct transcriptional target of LXR, a sterol-sensing nuclear receptor that triggers an “emergency response” to a lipid overload state. Activation of LXR induces the expression of genes involved in cholesterol efflux (*ABCA1* and *ABCG1*), limiting lipid uptake (*IDOL*), and promoting triglyceride-rich lipoprotein formation (*SREBP1C* and *SCD1*)^8^. On the other hand, the sterol regulatory element-binding proteins (SREBPs) are master regulators of sterol metabolism, directly activating the expression of genes involved in cholesterol and fatty acid biosynthesis^9^. Although all SREBP isoforms can influence a large repertoire of genes at extreme perturbations, it is well established that SREBP1c preferentially activates genes involved in fatty acid biosynthesis, whereas SREBP2 influences cholesterol biosynthetic machinery^10^. Consistent with unique epistatic relationship between various SREBPs, loss of SREBP2 in mouse liver reduces SREBP1c and triglyceride levels in addition to impacting cholesterol stores^11^. Despite their unique activation signature both isoforms appear to bind similar DNA response elements and to partner with common transcriptional coactivators, including Nuclear Transcription Factor Y (NFY) and SP1 (ref. ^12,13^).

In this work, we outline a role for hnRNPs in regulatory circuits controlling sterol homeostasis. Liver-specific deletion of the hnRNP *Raly* lowers serum cholesterol and hepatic lipid content. By mapping RALY-binding sites and utilizing unbiased chromatin interrogation techniques, we show preferential binding patterns of RALY at gene promoter regions and decipher its cooperative interactions. Intriguingly, we find that RALY binds at the *Srebp2* but not *Srebp1* promoter region, and show that it interacts directly with NFY to influence transcription of cholesterogenic genes. Our work offers insights into mechanisms orchestrating selective promoter activation and a model by which hnRNPs can impact metabolic disease states.

## Results

### Ablation of Raly impacts cholesterol biosynthetic genes

**Fig. 1:**
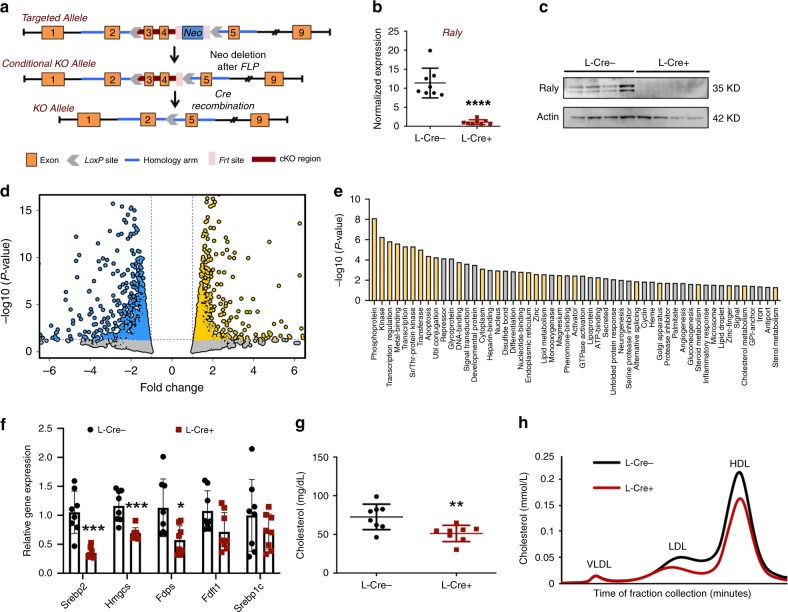
Loss of RALY reduces Srebp2 and specifically enriches for metabolic pathways. **a** Schematic of Raly conditional knockout strategy. **b** Gene expression of RALY in liver from chow-fed mice (*n* *=* 8 per group). **c** Protein level of RALY in liver from chow-fed mice (*n* *=* 8 per group). **d** Volcano plot of RNA-seq results from liver with and without RALY (*n* *=* 4 per group). **e** Enriched functional terms from RNA-seq of differentially regulated genes from mouse liver. Yellow bar indicates functional keyword is also enriched in liver SREBP2 ChIP. **f** Gene expression in mice liver on chow diet (*n* *=* 8 per group). **g** Total serum cholesterol levels isolated from chow-fed mice (*n* = 8 per group). **h** Cholesterol levels in pooled fractionated serum from mice in **g**. All data are mean ± SD. *P < 0.05; **P < 0.01, and ***P < 0.001, using two-tailed Student’s *t*-test.

To gain insights into the contribution of hnRNPs in metabolic disease, we generated mice with LoxP sites flanking exons 3 and 4 of *Raly* (Fig. 1a, Supplementary Fig. 1). Administration of a Cre or control adenovirus to *Raly*^flox/flox^ primary murine hepatocytes resulted in the ablation of RALY transcript and protein levels (Supplementary Fig. 2a, b). Since RALY is one of the few hnRNPs linked to human lipid traits, and since our previous studies have shown that disruption of the *LeXis*-RALY axis perturbs cholesterogenic gene expression, we sought to determine the effect of genetic deletion of *Raly* on *Srebp2* (official gene symbol Srebf2) gene expression. Deletion of *Raly* from primary murine hepatocytes led to a significant reduction of Srebp2 and its target genes involved in cholesterol biosynthesis, including Hmgcr (Supplementary Fig. 2c). Surprisingly, the deletion of *Raly* from mouse hepatocytes also led to a significant reduction in Srebp1c, the isoform responsible for triglyceride biosynthesis (Supplementary Fig. 2c). We confirmed reduced protein levels of a nuclear SREBP2 and a number of targets, including 3-Hydroxy-3-Methylglutaryl-CoA Synthase (HMGCS) and Farnesyl Diphosphate Synthase (FDPS) (Supplementary Fig. 2d, e). To explore the contributions of *Raly* in liver metabolism, we generated liver-specific *Raly* knockout mice (referred to as L-*Raly*KO while *Raly*^flox/flox^ Cre-negative littermates are controls). Quantitative PCR (qPCR) analysis and western blotting confirmed significant and robust decrease in RALY in liver following Cre recombination (Fig. 1b, c). Previous studies have shown that at least a subset of hnRNP complexes influence gene expression in a non-discriminate fashion^14^. To better define the range of RALY activities in liver, we performed unbiased transcriptional profiling of livers from L-*Raly*KO and control mice on chow diet (Fig. 1d). Analysis of differentially regulated genes showed a significant and strong enrichment of lipid metabolic and related pathways (Fig. 1e), as well as substantial overlap with pathways known to be modulated by SREBP2 (Fig. 1e)^15^. qPCR showed a significant reduction in expression of Srebp2 and its target genes in L-RalyKO mice (Fig. 1f). There was also a trend to reduced Srebp1c expression although did not reach significance (fasting mice). Consistent with gene expressing results loss of RALY was associated with a reduction in serum cholesterol level (Fig. 1g). Lipid fractionation analysis revealed a reduction in low-density lipoprotein (LDL) and high-density lipoprotein fractions the predominant circulating pool in chow-few mice (Fig. 1h). Taken together, these results suggest that hnRNPs can regulate the activity of specific gene expression programs and that the effects of RALY on cholesterogenesis are non-redundant.

### Liver-specific deletion of Raly reduces hepatic sterol content

**Fig. 2:**
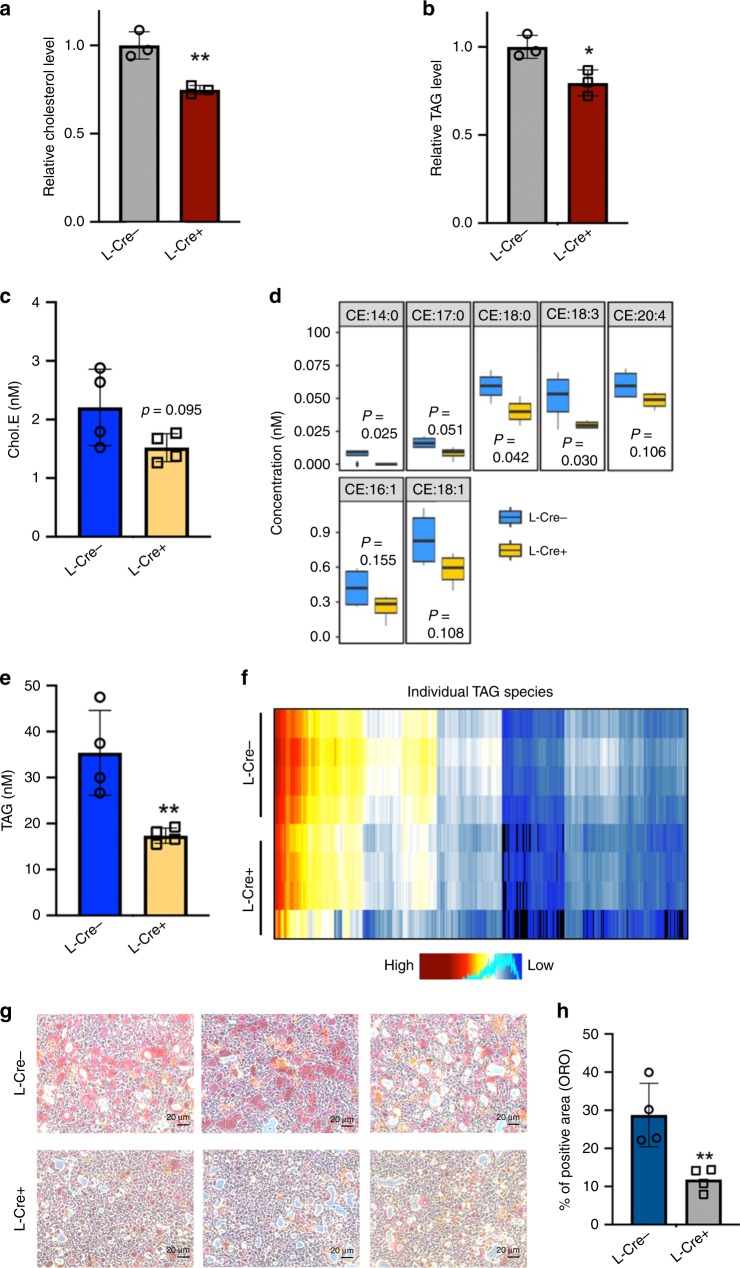
Liver-specific deletion of RALY alters hepatic lipid content. **a**, **b** Cholesterol level **a** and triglycerides level **b** from primary hepatocytes relative to baseline levels (normalized to cell number). **c**, **d** Lipidomics investigation of cholesterol ester (CE) species from liver from L-Raly knockout mice and controls (*n* = 4 per group). **e**, **f** Lipidomics investigation of triglycerides species from liver from L-Raly knockout mice and controls (*n* = 4 per group). Each line on heat map represents an individual triglyceride species of 411 different triglycerides detected (four mice per group). **g** Oil Red O (ORO) staining of liver from NASH diet-fed mice (scale bar, 20 µm). **h** Quantification of positive ORO staining area from done with automated image j detection. Values are shown as means ± SD. *P < 0.05 and **P < 0.01, using two-tailed Student’s *t*-test.

We noted that loss of *Raly* from hepatocytes was associated with a decrease in cellular cholesterol and triglyceride content (Fig. 2a, b). To better define the contributions of RALY on hepatic lipid composition, we performed unbiased shotgun lipidomics on mouse liver comparing L-*Raly*KO and controls. We observed that vast majority of lipid species were unchanged with the exception of hepatic cholesterol and triglyceride content (Fig. 2, Supplementary Fig. 3). Examination of cholesterol esters showed a reduction in most species in L-*Raly*KO although only a subset reached statistical significance (Fig. 2c, d). Similarly triglyceride content was significantly reduced in L-*Raly*KO livers (Fig. 2e, f). We observed no changes in the expression of genes involved in lipolysis (Supplementary Fig. 4). Intriguingly, these results partially phenocopy the chow-fed *Srebp2* liver-specific knockout mice, that exhibit mildly reduced liver cholesterol content as well altered SREBP1c and triglycerides levels^11^. These results are also consistent with unique epistatic relationship between various SREBPs. Taken together, the lipidomics findings reinforce the gene expression results and support the notion that RALY may be affecting sterol metabolism through interaction with the *Srebp2* pathway. To more thoroughly investigate the contributions of RALY in chronic lipid abundance states, we fed L-*Raly*KO mice and controls a diet known to induce sterol accumulation and Non-Alcoholic Steatohepatitis (NASH). After 9 weeks of feeding, L-*Raly*KO showed significant reduction in Oil Red O staining (Fig. 2g, h). These results demonstrate that in a preclinical disease model the hepatic loss of RALY partially protects against lipid overload.

### DNA binding of RALY enriches for metabolic regulators

**Fig. 3:**
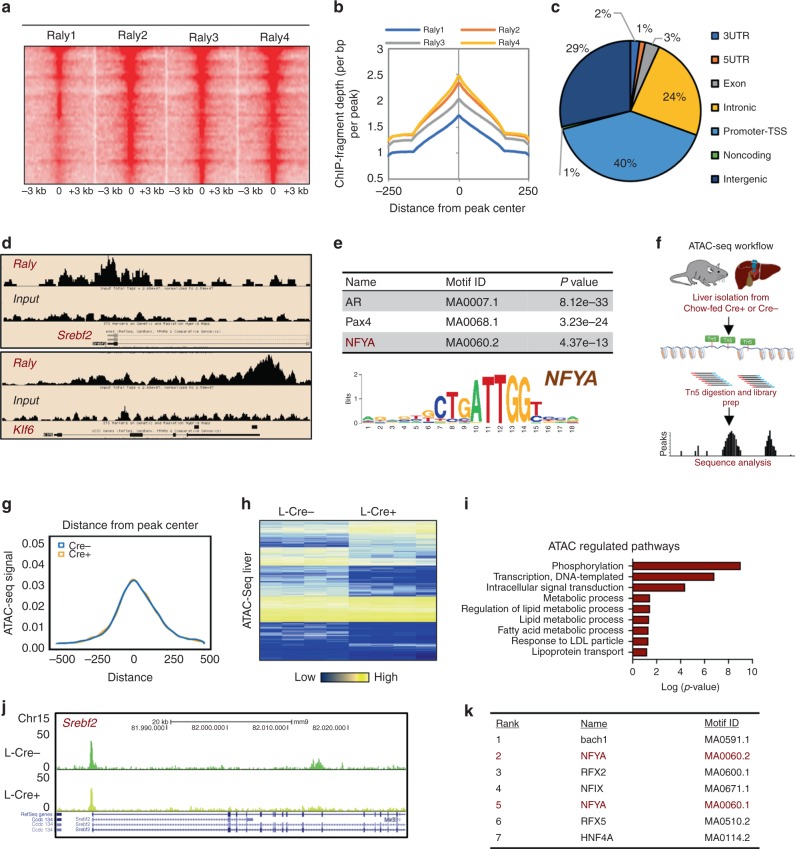
Interrogation of the RALY cistrome and chromatin dynamics enriches for promoter binding and metabolic coregulators. **a** Heat map of reproducible counts centered around a gene transcription start site for RALY ChIP-seq samples in hepa1-6 cells (*n* = 4 per group). **b** ChIP fragment depth enrichment for RALY across samples. **c** Pi chart showing the binding pattern of RALY according to the location of a given peak. **d** Representative RALY ChIP-seq profiles at *Srebf2* and *Klf6* loci. **e** Motif analysis surrounding called peaks from RALY ChIP-seq in hepa1-6 cells. **f** Schematic of ATAC-seq workflow. **g** Metagene representation of the mean ATAC-seq signal. ATAC-seq was performed from mice liver with Raly f/f L-Cre+ or Cre− (*n* = 4 per group). **h**, **i** Heat map shows the differential enrichment of ATAC peaks of Cre− or Cre+ livers and Gene ontology analysis. **j** Representative ATAC-seq heat map in liver with Raly f/f L-Cre+ or Cre− at the *Srebf2* locus. **k** Motif analysis showing top enriched factors at differentially regulated sites from Raly f/f L-Cre+ or L-Cre− liver.

Our previous studies have shown that *LeXis* and RALY are almost exclusively present in the cell nucleus in association with chromatin and that *LeXis* may be impacting cholesterogenic gene expression through transcriptional mechanisms^7^. Thus, we hypothesized that RALY may also be impacting *Srebp2* levels through nascent transcript production. To gain better insight as to how RALY may regulate gene expression, we mapped genome-wide RALY-binding sites. We performed chromatin immunoprecipitation sequencing (ChIP-seq) to assess RALY DNA binding in murine hepatocytes and identified a total of 2950 RALY peaks that were independent identified by the same peak calling algorithm in at least two independent samples (Fig. 3a). Global analysis of fragment distribution around the peak summit showed overall agreement between samples and a broad peak contour profile spanning ~500 bps, a feature often associated with coregulators (Fig. 3b). Interestingly, RALY showed strong enrichment for promoter binding but was also in enriched in other parts of the genome, including intronic and intergenic regions (Fig. 3c, d). Furthermore, unbiased peaking calling showed that RALY bound the *Srebp2* promoter region but not the *Srebp1* promoter (Fig. 3d, Supplementary Fig. 5). These results reinforce the notion that RALY primarily affects Srebp2 and that perturbations in hepatic triglycerides content are likely a downstream consequence of reduced SREBP2 activity. In addition, motif discovery analysis of RALY-bound peaks identified the transcription factor NFY as a highly enriched motif (Fig. 3e). NFY is a promoter-binding transcription factor (formed with trimeric complex of NFYA, NFYB, and NFYC with all subunits required for proper function) with an established role in mediating SREBP responses^16^. Taken together, these results hint that RALY may be influencing cholesterogenesis by modulating collaborative interactions with transcriptional coactivators at the *Srebp2* promoter.

A number of studies have shown that noncoding RNA–protein interactions can regulate gene activities by modulating a number of histone modifications at target genes^17,18^. To explore the possibility that RALY may influence gene expression by altering epigenetic states, we performed Assay for Transposase-Accessible Chromatin using sequencing (ATAC-seq)^19^, a method that allows unbiased interrogation of chromatin architecture, on livers from L-*Raly*KO and controls (Fig. 3f). Loss of RALY in mouse liver did not globally alter chromatin accessibility (Fig. 3g) arguing against the idea that RALY may be impacting gene expression by impacting histone modifiers, such as polycomb repressive complex proteins or histone deacetylase^20^. In addition to directly interrogating changes in nucleosome rearrangements, ATAC-seq allowed us to infer transcriptional activity by examining changes in enhancer landscapes with targeted perturbations. Although globally most ATAC peaks did not change between controls and L-*Raly*KO samples, a number of peaks were differentially regulated (Fig. 3h). Intriguingly, many of these peaks clustered near genes involved in lipid metabolism (Fig. 3i, j, Supplementary Fig. 6). Furthermore, motif analysis of these differentially altered peaks showed specific enrichment for number of transcription factors known to impact metabolic regulation, including NFY (Fig. 3k). Taken together, the above results suggest that RALY may be regulating cholesterol metabolism by impacting the coactivator NFY at the *Srebp2* gene.

### RALY is required for NFY-dependent activation of Srebp2

**Fig. 4:**
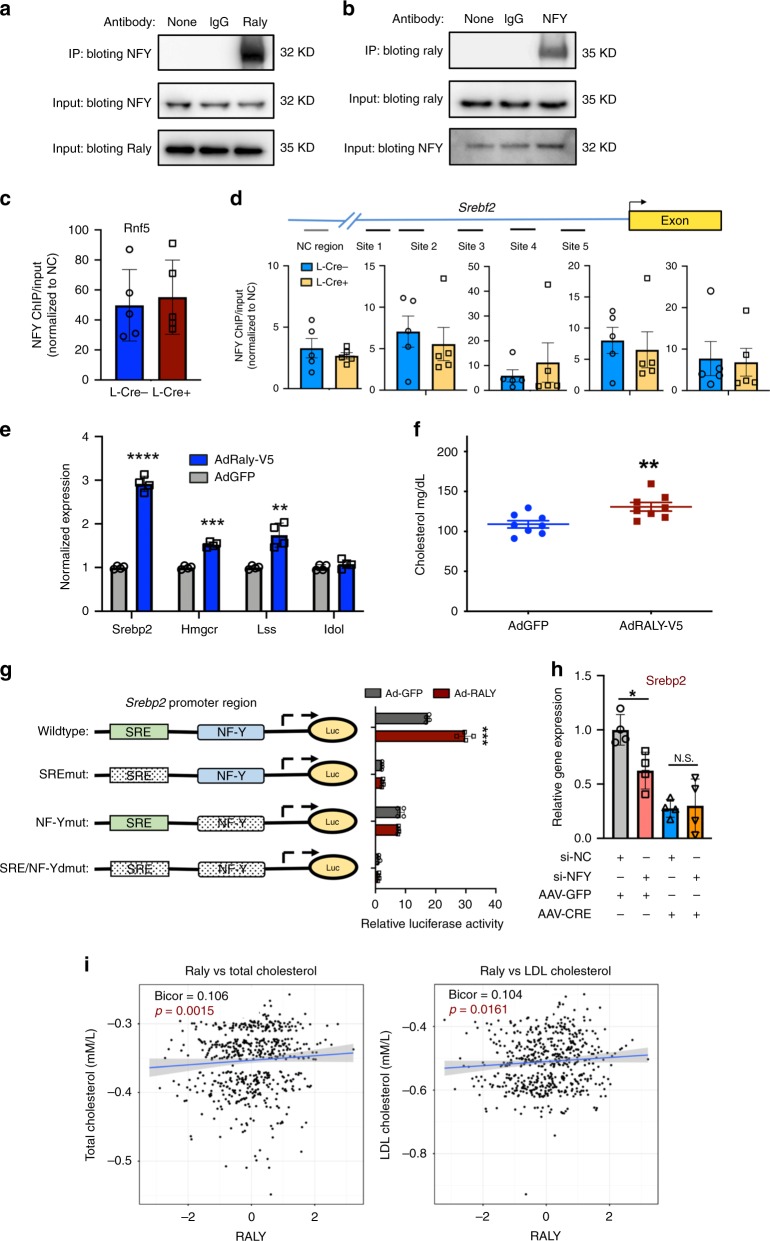
The coactivator NFY is required for the transcriptional effects of RALY on cholesterogenesis. **a**, **b** Western blot from Co-IP studies performed in hepa1-6 cells. **c** NFY ChIP-qPCR of the positive control NFY gene done from Raly f/f L-Cre+ or L-Cre− livers (*n* = 5 per group). **d** ChIP-qPCR on Srebf2 promoter using various primers around NFY sites from ChIP in **c**. Data were normalized to negative control region; (*n* = 5 per group). Data are mean± SEM. **e** Gene expression results from hepa1-6 cells treated with adenovirus GFP or RALY (*n* = 4 per group). **f** Serum cholesterol levels from chow-fed mice treated with adenovirus GFP or RALY harvested after 6 days of injection. Data mean± SEM; (*n* = 8 per group). **g** Luciferase promoter assays of Srebp2 promoter region performed in hepa1-6 cells and with GFP or RALY overexpression. **h** Srebp2 gene expression after NFY knockdown in control or RALY-deficient primary hepatocytes (*n* = 4 per group); values are mean±SD. **i** Gene expression results from METISM study showing correlation of metabolic traits with RALY. Values are inverse normal transformed. *P < 0.05; **P < 0.01, ***P < 0.001, and ****P < 0.0001.

To clarify whether RALY directly interacts with transcriptional machinery at SREBP2, we performed co-immunoprecipitation (Co-IP) studies in murine hepatocytes. Pulldown of RALY enriches for NFY and vice versa confirming a robust interaction between the two factors (Fig. 4a, b). To clarify whether RALY is required for NFY binding at promoter regions, we performed ChIP of NFY in L-*Raly*KO or control livers. We confirmed NFY enrichment at its known target gene Rnf5 (Fig. 4c), as well as the SREBP2 promoter region (Fig. 4d). Our results suggest that NFY binding is minimally rearranged by the loss of RALY consistent with the idea that RALY does not act as a guide to facilitate NFY complex DNA binding, rather it works cooperatively with NFY at select sites to influence its transcriptional activity. To determine if RALY was sufficient to induce the expression of cholesterogenic genes, we overexpressed RALY using an adenoviral vector in a murine hepatocyte cell line (Supplementary Fig. 7). We found that cholesterogenic gene expression was increased in response to RALY expression (Fig. 4e). In addition, hepatic RALY overexpression increased serum cholesterol in chow-fed mice (Fig. 4f). To more thoroughly investigate the cooperative relationship between RALY and cis/trans promoter factors, we performed luciferase reporter assays with an *Srebp2* promoter construct (Fig. 4g). Adenoviral expression of RALY in hepatocytes enhanced wild-type *Srebp2* promoter-driven luciferase activity, but failed to increase luciferase activity when either NFY or SRE sites were mutated (Fig. 4d). These results reinforce the idea that RALY is a coactivator that requires the binding of canonical transcriptional factors at the *Srebp2* promoter including SREBP itself. To better clarify the epistatic relationship between NFY and RALY, we performed a knockdown of NFY in RALY-deficient cells. Our results show that reduction of NFY on a *Raly*-deficient background no longer alters Srebp2 expression (Fig. 4h, Supplementary Fig. 8). These results suggest that RALY is required for NFY-dependent transcription of SREBP2 and is consistent with notion, and RALY and NFY work cooperatively to influence gene expression.

To better explore the relationship between hnRNPs and human lipid traits, we analyzed expression data from the *METISM* cohort^21^. Interestingly, expression of RALY was positively associated with a number of metabolic, traits including total and LDL serum cholesterol levels though the strength of association was modest (Fig. 4i). Importantly, other hnRNPs, including ones that contribute to transcriptional control mechanisms (hnRNP K), were not significantly associated with total cholesterol or LDL cholesterol in this cohort (Supplementary Fig. 9). Although hnRNPs are known to form complexes with one another, these observations are consistent with the idea that individual hnRNPs may play functional roles favoring specific pathways

## Discussion

Previous work has identified important roles for hnRNPs in neurodegenerative disease, most prominently amyotrophic lateral sclerosis and frontotemporal lobar degeneration^1,2^. Our work expands the contributions of hnRNPs in health and disease by showing that a conserved hnRNP can help direct the fundamental metabolic regulatory circuits. In addition, our work offers insights into the precise molecular mechanisms that link hnRNP abnormalities with pathologic states. The functional versatility of hnRNPs is thought to stem from their ability to “dance with different partners” to impact diverse biologic process, such as RNA splicing, polyadenylation, export, and translation. Thus, there are potentially multiple ways by which hnRNPs abnormalities can lead to disease states. Defects in hnRNPs leading to stress granule changes and the accumulation of pathological inclusions are thought to be important in neurodegenerative states^22,23^. Our work shows that hnRNPs proactively participate in transcriptional control mechanisms regulating cholesterol homeostasis and that loss of a single hnRNP (RALY) influences hepatic lipid stores. It should be noted that our work does not exclude the possibility that RALY may play other functional roles or influence gene regulation though multiple mechanisms. In addition, RALY may be impacting transcript splicing or other redundant functions that may be masked in our knockout model or perhaps more critical in other tissues or cell types. Intriguingly, we find that RALY has multiple isoforms and it is unclear whether different isoforms perform different functions.

It is well established that the predominantly cholesterogenic SREBP2 and lipogenic SREBP1 transcription factors may be differentially processed depending on the environment cues. However, both isoforms are capable of binding diverse SREs and collaborate with common coactivators despite possessing distinct transcriptional effects once in the nucleus. In addition, it is unclear how generic collaborative partners, such as NFY and SP1 are capable of turning on some but not all their target genes in response to specific environmental cues. The characterization of RALY offers some important clues into the selective promoter activities of different SREBPs. The fact that RALY binds the promoter of one isoform (*Srebp2*) and not the other hints that spatial collaborative interactions may favor a specific gene activation signature. The finding that RALY interacts with the SREBP coactivator NFY also raises a number of intriguing questions. How do hnRNPs influence some genes but not others? and what is the biochemical basis for their interactions? can RALY directly collaborate with other transcriptional regulators besides NFY? Future studies will provide more insights into these questions.

## Methods

### Study approval

All experiments were approved by the UCLA Institutional Animal Care and Research Advisory Committee and performed in strict accordance with the recommendations in the Guide for the Care and Use of Laboratory Animals of the National Institutes of Health. Please see supplemental materials for detailed methods.

### Animals

All animals used in the study were in C57BL/6 background. Our study used male mice unless otherwise specified. Mice were fed chow diet (Research Diet) and housed temperature-controlled room under a 12-h light/12-h dark cycle and pathogen-free conditions. *Raly*^flox/flox^ mice were generated by Cyagen using the strategy outlined in Fig. 1a. To the generated RALY liver-specific knockout mice and littermate controls, we treated *Raly*^flox/flox^ with adeno-associated virus (AAV) with TBG promoter (AAV8.TBG.Cre) or (AAV8.TBG.GFP; green fluorescent protein) purchased from Penn Vector Core. AAV administered intraperitoneal injection at dose of 5 × 10^11^ GC per mice. Mice were euthanized 4 weeks after AAV injection. Liver tissues were frozen in liquid nitrogen and stored at −80 °C or fixed in 10% formalin. Blood was collected by retro-orbital bleeding, and the plasma was separated by centrifugation. Plasma lipids were measured with the Wako L-Type TG M kit, the Wako Cholesterol E kit. All animal experiments were approved by the UCLA Institutional Animal Care and Research Advisory Committee.

### Cells culture

Mouse primary hepatocytes were isolated as previously described and cultured in William’s E medium with 5% bovine serum albumin (BSA)^24^. Hepa1-6 cells were originally obtained from ATCC and cultured in Dulbecco's Modified Eagle Medium (DMEM) medium with 10% fetal bovine serum (FBS). Adenovirus studies were performed as previously described^7^. RALY was cloned from mouse cDNA using a gateway cloning system and into the pAd/CMV/V5-DEST Gateway vector by LR recombination according to the manufactures guidelines. NFY knockdown was done in primary hepatocytes using short interfering RNA against nfya (Dharmacon^TM^ catalog number LQ-065522-00-0005). Transfection was proceed using DharmaFECT^TM^ 4 transfection reagent (Dharmacon^TM^) according to the manufacturers recommendation. Cells were collected for RNA isolation or protein extraction 48 h after transfection.

### Gene expression analysis and immunoblot analysis

Total RNA was isolated using TRIzol reagent (Invitrogen) and reverse transcribed using a homemade RT, as we previously described^25^. cDNA was quantified by real-time PCR using SYBR Green Master Mix (Diagenode) on BioRad Real-time PCR instrument. Gene expression levels were determined by using a standard curve. Each gene was normalized to the housekeeping gene 36B4. For immunoblot analysis, whole cell lysate or tissue lysate was extracted using RIPA lysis buffer (Boston Bioproducts) supplemented with complete protease inhibitor cocktail (Roche). For SREBP2 proteins immunoblot analysis, nuclei from primary hepatocytes were prepared using Subcellular Protein Fractionation Kit for Cultured Cells (78840, Thermo Scientific). Proteins were diluted in Nupage loading dye (Invitrogen), heated at 95 °C for 5 min, and run on 4–12% NuPAGE Bis-Tris Gel (Invitrogen). Proteins were transferred to hybond ECL membrane (GE Healthcare) blocked with 5% milk (or 5% BSA for anti-SREBP2) to quench nonspecific protein binding and blotted with the indicated primary antibody. For complete listing of antibodies and primers please see Supplementary Table 1. Uncropped blots are provided in Supplementary Fig. 10.

### Dual luciferase assay

DNA transfection of Hepa1-6 cells was performed with LipofectamineTM 3000 (Invitrogen) according to user’s manuscript on 24-well plates with a cell density of 1.5 × 10^5^ cells/well. The cells were transfected with 200 ng of the *Srebp2* promoter firefly reporter plasmid, 50 ng of *Renilla* reporter plasmid (Promega), and 200 ng of nSREBP2 expression vector. At 12 h after transfection, the cells were cultured in DMEM supplemented with 1% (v/v) FBS and adenovirus was administered. After 24 h, assays for both luciferase and Renilla activities were performed. The reporter activities were expressed as the relative firefly luciferase activity/Renilla luciferase activity.

### RNA-seq

Libraries for RNA-Seq were prepared with KAPA Stranded RNA-Seq Kit on RNA isolated from livers of chow diet feeding mice with AAV-GFP or AAV-Cre transduction. The data were sequenced on Illumina HiSeq 3000 for a pair-end 150 bp read run. Data quality check were done on Illumina SAV. Demultiplexing was performed with Illumina Bcl2fastq2 v 2.17 program. RNA-seq reads were aligned with TopHatv2.0.2 to the mouse genome, version mm9 25. Transcripts were assessed and quantities were determined by Cufflinks v2.0.2, using a GTF file based on Ensembl mouse NCBI37. Comparison expression levels were made using Fragments Per Kilobase of transcript per Million (FPKM) values using Cuffdiff from the Cufflinks package 26.

### NASH diet feeding and Oil Red O staining

Twelve-week-old male mice were fed with diet composition of 60 kcal% fat with 0.1% methionine and no added choline (A06071302, Research Diets) for 9 weeks. Oil Red O histochemistry staining were performed using frozen sections from mouse liver. Briefly, frozen sections were fixed in 10% neutral formalin for 10 min, followed by treatment in 60% isopropanol for 5 s and then staining in Oil Red O working solution (O-0625, Sigma Aldrich) for 15 min. This procedure was followed by washing with 60% isopropanol for 5 s and then water 1 min. Finally, sections were contained with Mayer’s Hematoxylin for 3 min.

### Lipidomics

A total of 50–100 mg of frozen liver are homogenized in the Omni Bead Ruptor Elite with 2 mL homogenizer tube system (Omni, 19-628D). Samples are homogenized in cold phosphate-buffered saline (PBS) for 3 cycles of 10 s each at 5 m/s with a 10 s dwell time between cycles. A total of 3–6 mg of homogenized material are applied to a modified Bligh and Dyer extraction^26^. Prior to biphasic extraction, a 13 lipid class Lipidyzer Internal Standard Mix is added to each sample (AB Sciex, 5040156). Following two successive extractions, pooled organic layers are dried down in a Genevac EZ-2 Elite. Lipid samples are resuspended in 1:1 methanol/dichloromethane with 10 mM ammonium acetate and transferred to robovials (Thermo 10800107) for analysis.

Samples are analyzed on the Sciex Lipidyzer Platform for targeted quantitative measurement of 1100 lipid species across 13 lipid sub-classes. Differential Mobility Device on Lipidyzer is tuned with SelexION tuning kit (Sciex 5040141). Instrument settings, tuning settings, and MRM list available upon request. Data analysis performed on Lipidyzer software. Quantitative values are normalized to milligrams of material used.

### Chromatin immunoprecipitation

ChIP experiments were performed as we previously described with exception of a few changes^25^. For RALY ChIP, 20 million cells were used for each sample and four replicates were performed for each group. Sonication was performed using a M220 Focused-ultrasonicator (Covaris) according to the manufacturer’s protocol (10 min for cells), and chromatin was immunoprecipitated with 4 μg antibodies against RALY (Ab170105, Abcam) overnight at 4 °C. ChIP-Seq libraries were prepared using the Kapa LTP Library Preparation Kit (Kapa Biosystems). ChIP-Seq was performed as described^27^. Bowtie2 was used for alignment and we identified Raly enriched peaks in using by Homer findPeaks^28^ with FDR < 0.01. The called peaks were subsequently used for identifying the motifs enrichment by PscanChIP^29^. For NFY ChIP, chromatin samples from mice livers were prepared using truChIP Chromatin Shearing Tissue Kit (Covaris) according to manufacturer’s recommendation. Sonication was performed using a M220 Focused-ultrasonicator (Covaris) according to the manufacturer’s protocol for 12 min, and chromatin was immunoprecipitated with 6 µg antibodies against NFYA subunit (sc-17753×, Santa Cruz Biotechnology) overnight at 4 °C. The ChIP samples were analyzed by real-time PCR using primers listed in Supplementary Table 1. All values obtained were normalized to the primers of negative control region.

### ATAC-seq

ATAC-seq was optimized in liver after several modifications from original Buenstero protocol^19^. A total of 100 mg of frozen liver were grinded to fine powder using cellcrusher and 1 mL of ice cold nuclei isolation buffer was added (20 mM Tris-HCl, 50 mM EDTA, 5 mM spermidine, 0.15 mM spermine, 0.1% mercaptoethanol, 40% glycerol, pH 7.5, mM EGTA, and 60 mM KCl). After 5 min of cooling on ice, cell suspension was filtered through Miracloth (Calbiochem) followed by centrifugation at 1100 × *g* for 10 min at 4 °C. Pellet was resuspended with 50 µL RSB buffer (10 mM Tris-HCl, 10 mM NaCl, 3 mM MgCl2, and pH 7.4) followed by centrifugation at 500 × *g* for 5 min at 4 °C and resuspension in PBS. A total of 75,000 nuclei pellet were used for transposase reaction. The rest of the protocol followed that described by Buenstero. ATAC-Seq libraries were prepared using the Nextera Tn5 Transposase and DNA library preparation kit (Illumina) as described^19^. Libraries were single-end sequenced (50 bp) on an Illumina HiSeq 2000. Reads were mapped to the mouse genome (NCBI37/mm9) using Bowtie2 and were removed from the subsequent analysis if they were duplicated, mapped to mitochondrial genome, or aligned to unmapped contiguous sequences. Peak calling was performed using MACS2. The reads were converted to reads per thousand base pairs peak per million mapped reads (RPKM) by dividing by the total number of reads per sample. The average RPKM from four replicates was used to quantify the accessibility across all called peaks.

### Statistical analysis

A non-paired Student’s *t*-test was used to determine statistical significance, defined at *P* < 0.05. For multiple group experiments analysis of variance was used followed by multiple group analysis. Unless otherwise noted, error bars represent standard deviations. Experiments were independently performed at least twice. Sample size is based on statistical analysis of variance and prior experience with similar in vivo studies.

### Reporting summary

Further information on research design is available in the Nature Research Reporting Summary linked to this article.

## Supplementary information

Supplementary Information

Reporting Summary

## Data Availability

Data that support the findings of this study have been deposited in GEO with the following accession number GSE133166. All other relevant data supporting the key findings of this study are available within the article and its Supplementary Information files or from the corresponding author upon reasonable request. The source data underlying Figs. 1b, f–h, 2a–e, h, and 4c–h, and Supplementary Figs. 2a, c, 3 and 4 are provided as a source data file. A reporting summary for this article is available as a Supplementary Information file.

## Source data

Source Data
