# Supplementary material for: Collaborative interactions of heterogenous ribonucleoproteins contribute to transcriptional regulation of sterol metabolism in mice
Source: Nat Commun. 2020 Feb 20;11:984. doi: 10.1038/s41467-020-14711-4 (PMC7033216; doi:10.1038/s41467-020-14711-4)
Supplement: Supplementary file 1 — Supplementary Information [file 41467_2020_14711_MOESM1_ESM.pdf]

Supplementary Material

Collaborative interactions of heterogenous ribonucleoproteins contribute to transcriptional regulation of sterol metabolism in mice

Zhengyi Zhang et al.

Supplementary Table 1

Antibody	Company	Cat#	Utilization	Dilution
anti-RALY	Abcam	ab170105	Western blot, ChIP	1:1000
Anti-NFYA	Santa Cruz Biotechnology	sc-17753X	Western blot, ChIP	1:2000
Anti-NFYB	Abcam	ab111577	Western blot	1:1000
Anti-SREBP2	Millipore	MABS1988	Western blot	1:1000
Anti-PD1	cell signaling	3501S	Western blot	1:1000
Anti-LaminA/C	Santa Cruz Biotechnology	sc-376248	Western blot	1:800
Anti-tubulin	Abcam	ab185224	Western blot	1:1000
Anti-HMGCS	Abcam	ab137043	Western blot	1:1000
Anti-FDPS	Abcam	ab15385	Western blot	1:1000

Primer ID*	Sequence: Forward (5'-3')	Sequence: Reverse (5'-3')
ChIP_NC	TCCAGAAGAGGGCGTCAGAT	GGCTCAGTGGGTAAGAGCAC
ChIP Srebp2_Site1	GCTTCTTTTCCTGCTCAGGCT	GCTGTTGGGAAGCAGGTTA
ChIP Srebp2_Site2	TGTTCTGGGAACTCCACCTTT	TAGACCAGGCCAGCTTCAAAC
ChIP Srebp2_Site3	TGAAAGATGGTGGAGGACGC	AGAGACTGTGCTAAGTGACTGT
ChIP Srebp2_Site4	AAACAGTTGCTTAGCCAGCC	GGCAGTTGTTCTTAGGGGGT
ChIP Srebp2_Site5	CCCGCTATGCAAATCTGAGC	TTGTCAATGGGACCAGGCTT
ChIP Srebp2_Site6 (not enriched)	AGTCTTAGCCTGACCTGTCCT	CTTGTCAGAGGATTCAGACACCA
ChIP Srebp2_Site7 (not enriched)	AAAGGACGCAGTCGCATGT	ATGGAGAACCCAAGAAGGG
ChIP Srebp2_Site8 (not enriched)	TCCTTGGAACGTGGGAGTGGC	TGGCCAGTCTGACATCCAT

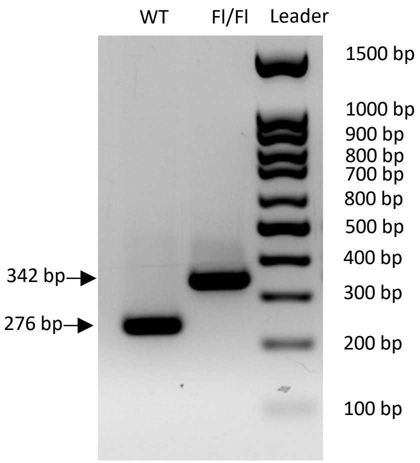

Supplementary Figure 1. ***Raly*** **flox/flox genotyping strategy.** Gel image of PCR genotyping showing WT and *Raly* flox/flox.

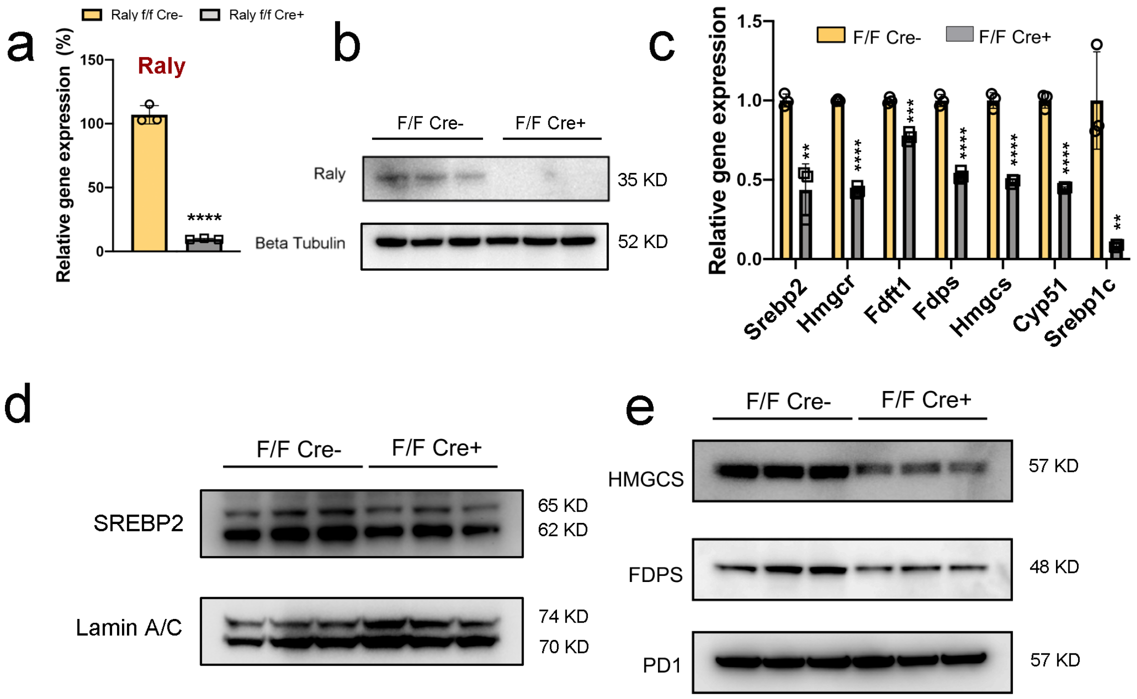

Supplementary Figure 2. Loss of Raly reduces SREBPs and target genes in primary hepatocytes. a Real-time PCR treated with GFP or Cre adenovirus. b Western blot analysis of RALY in mouse primary hepatocytes treated with GFP or Cre adenovirus. c Gene expression in mouse primary hepatocytes GFP or Cre adenovirus (n=3 per group). d Western blot analysis of nuclear SREBP2 in mouse primary hepatocytes treated with GFP or Cre adenovirus. e Western blot analysis of HMGCS and FDPS in mouse primary hepatocytes treated with GFP or Cre adenovirus. Values are shown as means ± SD. * $P < 0.05$; ** $P < 0.01$; *** $P < 0.001$ and **** $P < 0.0001$.

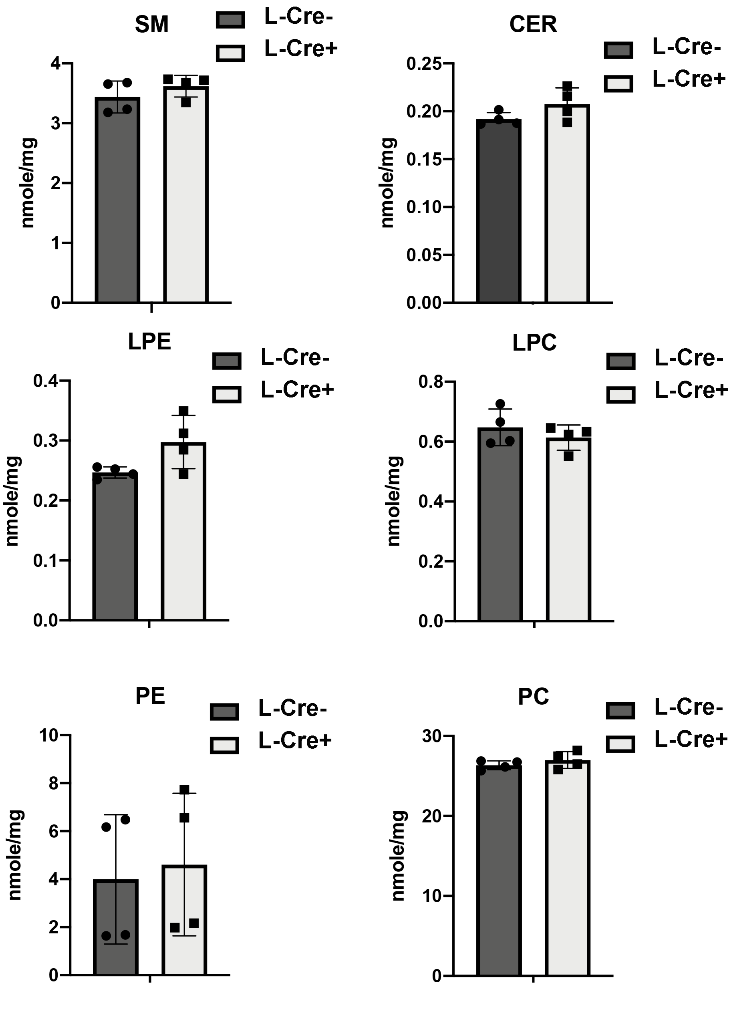

Supplementary Figure 3. Lipidomics analysis from mouse livers comparing *L-RalyKO* and controls. Species abbreviations are SM= Sphingomyelins, CER=Ceramides, LPE= Lysoglycerophosphoethanolamines, LPC=Lysoglycerophosphocholines, PE= Glycerophosphoethanolamines, and PC=Glycerophosphocholines

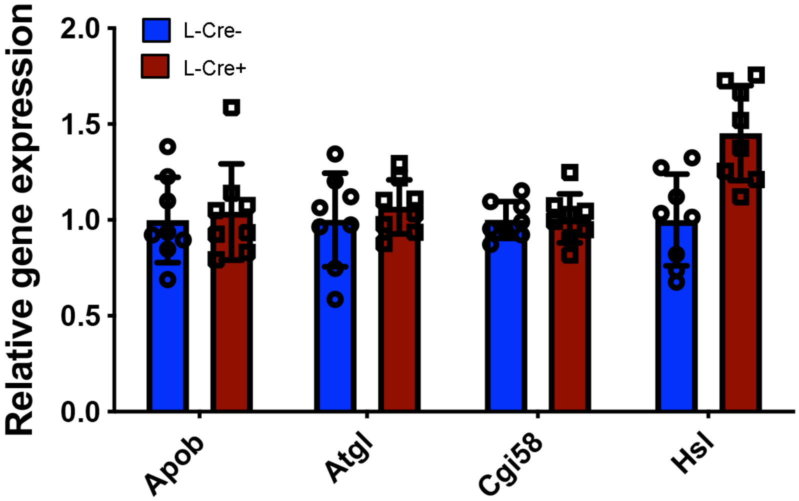

Supplementary Figure 4. Loss of RALY does not influence Lipolysis. Gene expression of in liver from chow-fed mice (*n* = 8 per group).

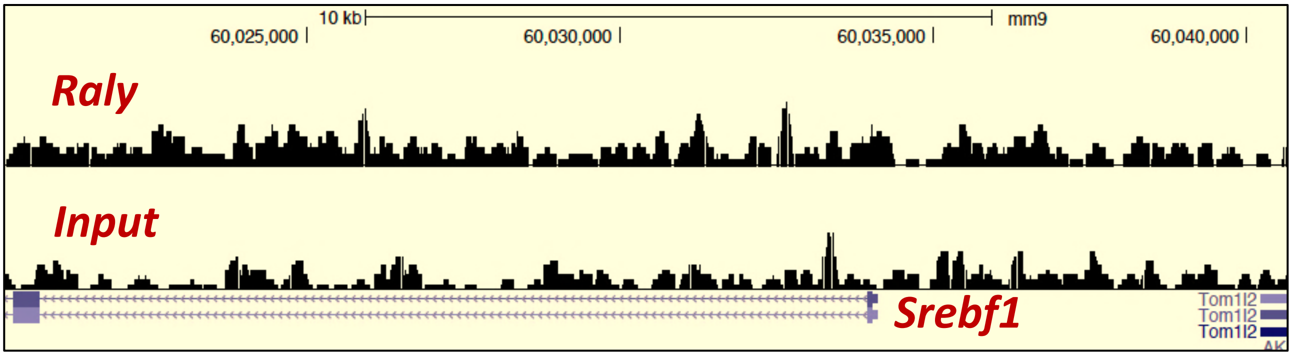

Supplementary Figure 5. RALY does not bind Srebf1 promoter. RALY ChIP-seq profiles at *Srebf1*.

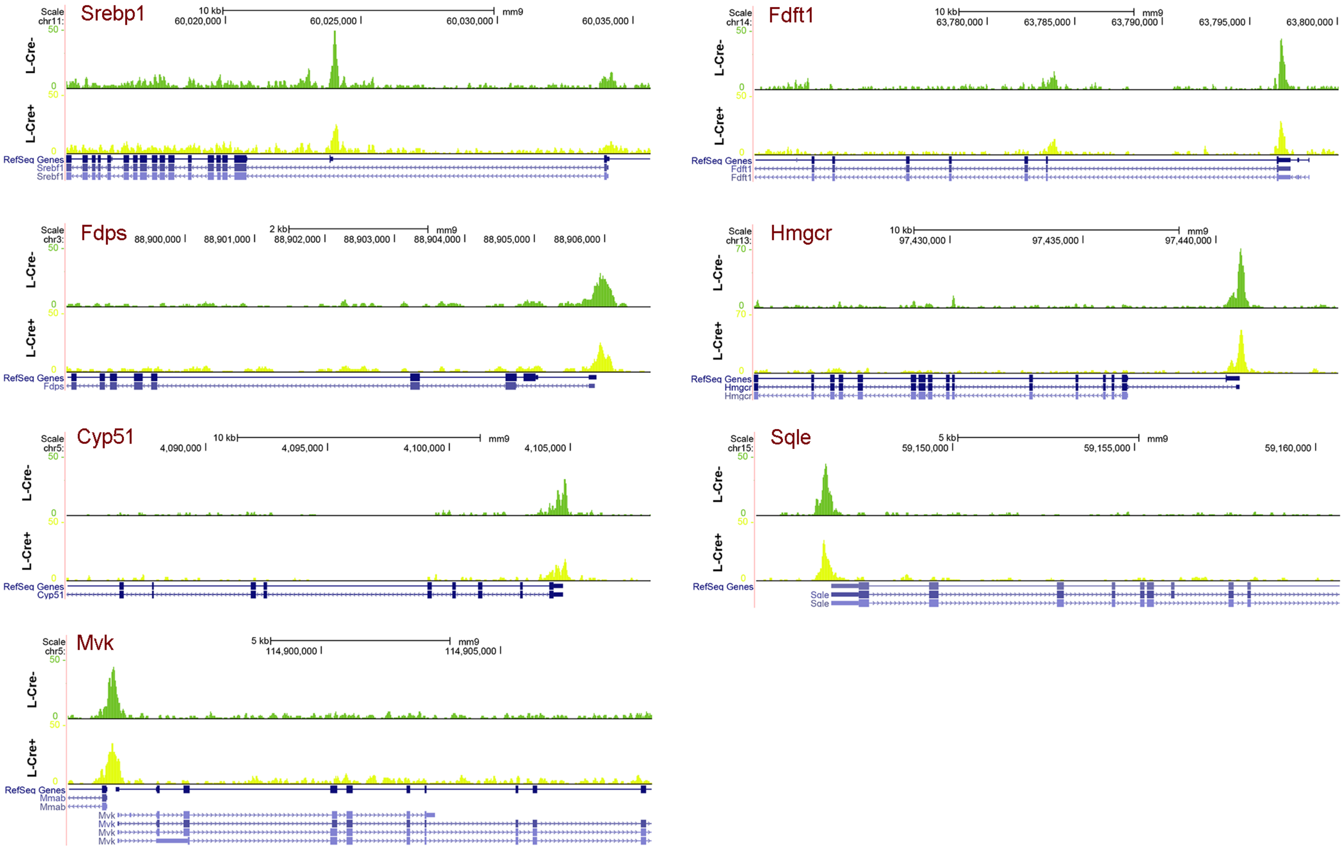

Supplementary Figure 6. ATAC-seq profiles at cholesterogenic genes as a consequence of loss of RALY. ATAC-seq profiles at *cholesterol biosynthetic genes.*

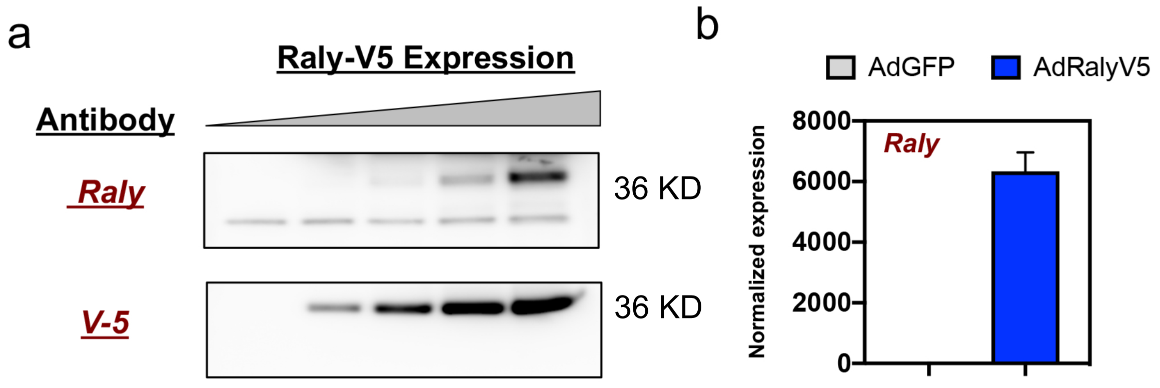

Supplementary Figure 7. Validation of RALY overexpression. a. Western blot from hepa1-6 cells for RALY and V5 at increasing RALY-V5 expression. b. Quantitative PCR of RALY for experiments performed in 4b.

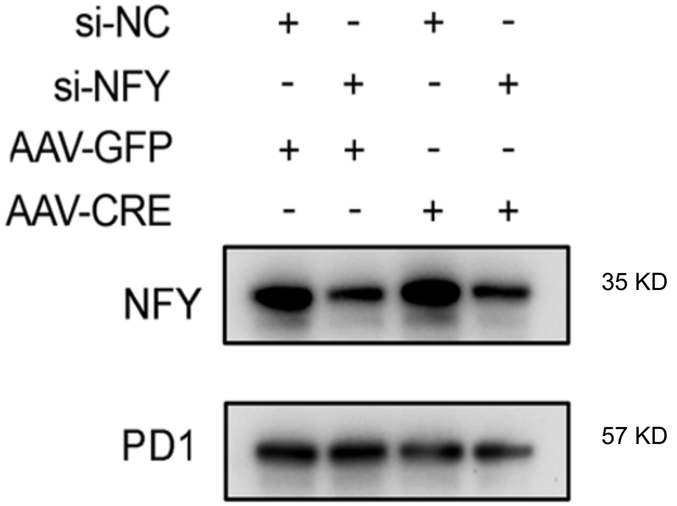

Supplementary Figure 8. Validation of NFY knockdown. a. Western blot of NFY from primary hepatocytes used in Fig 4h.

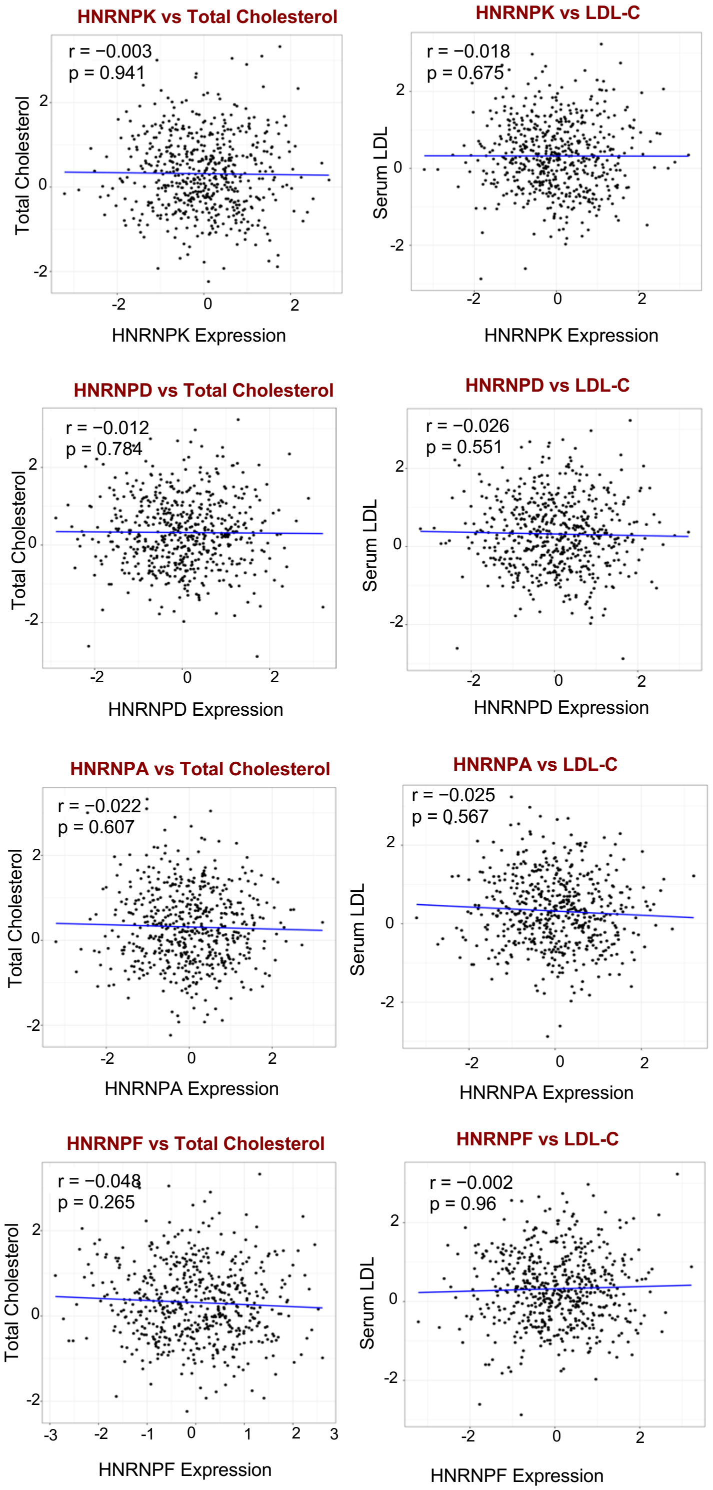

Supplementary Figure 9. Correlation of hnRNPs with Cholesterol Level. Expression results from METISM study showing correlation of metabolic traits with hnRNPs. Values are inverse-normal transformed.

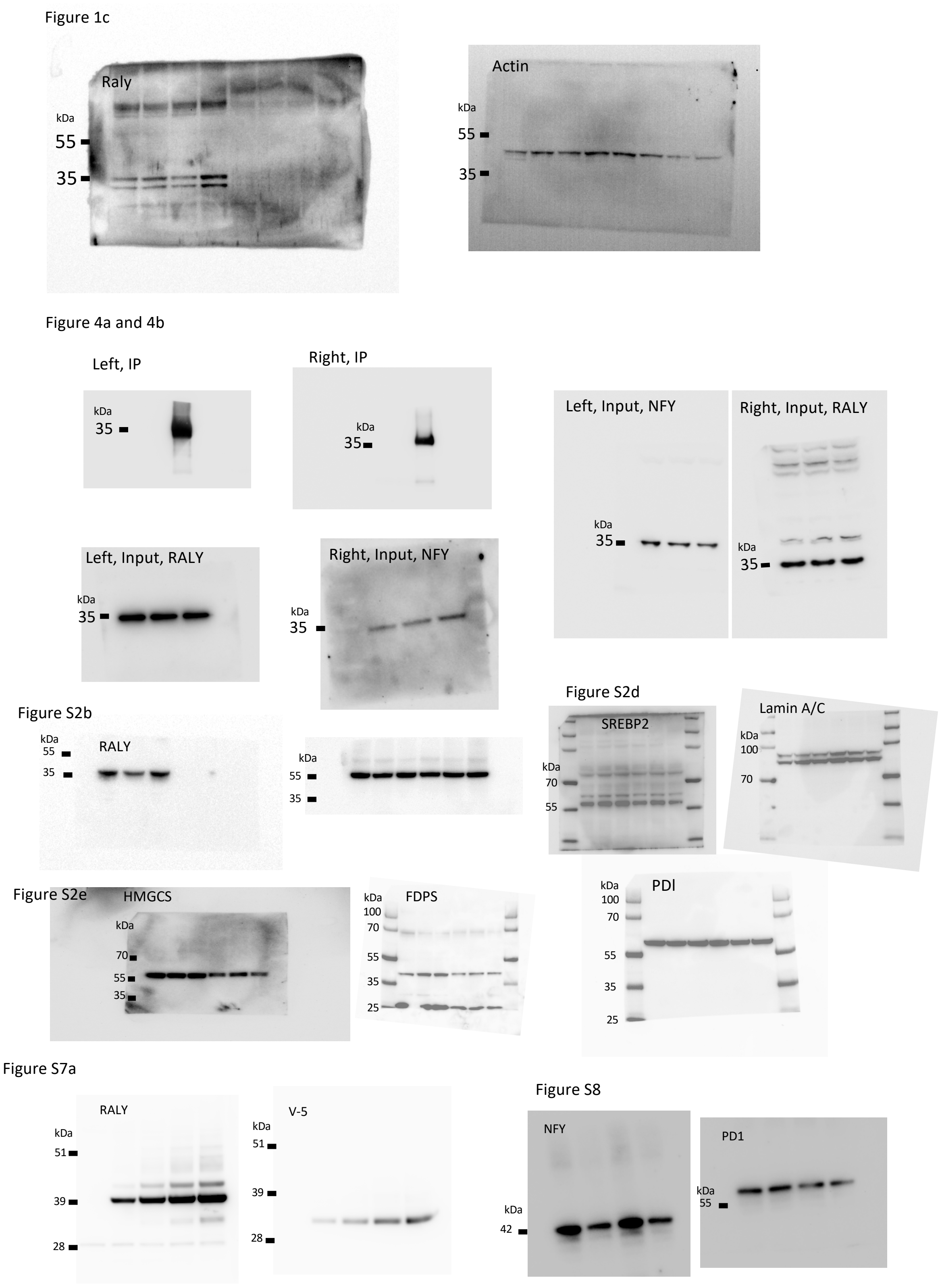

Supplementary Figure 10. Uncropped blots with molecular weight markers
